# Factors Associating with the Segmental Postural Control during Sitting in Moderate-to-Late Preterm Infants via Longitudinal Study

**DOI:** 10.3390/children8100851

**Published:** 2021-09-26

**Authors:** Noppharath Sangkarit, Wantana Siritaratiwat, Surussawadi Bennett, Weerasak Tapanya

**Affiliations:** 1 Research Center in Back, Neck, Other Joint Pain and Human Performance (BNOJPH), School of Physical Therapy, Faculty of Associated Medical Sciences, Khon Kaen University, Muang, Khon Kaen 40002, Thailand; noppharath.sa@kkumail.com (N.S.); surmac@kku.ac.th (S.B.); 2 Department of Physical Therapy, School of Allied Health Sciences, University of Phayao, Phayao 56000, Thailand; Weerasak.ta@up.ac.th

**Keywords:** moderate-to-late preterm, sitting, child rearing, segmental trunk control, environment

## Abstract

(1) Background: biological variables and particular child rearing practices could be linked to postural control and rates of sitting onset. The segmental Assessment of Trunk Control (SATCo) is currently used as an assessment of postural control with a specific segment on premature infants. However, the association between related factors and segmental trunk control during sitting development in preterm infants via longitudinal assessments is still limited. Objective: to investigate the associations between biological and child rearing factors and segmental trunk control during sitting in moderate to late premature birth from the age of 4 months to age of independent sitting attainment. (2) Methods: forty-two infants born between 32 and 36 weeks of gestation were recruited. Their segmental trunk control was assessed using the SATCo. Their related factors were recorded from the age of 4 months to early onset of independent sitting attainment. The generalised estimating equation (GEE) model was used to identify the association between related factors and the SATCo with a linear distribution. (3) Results: cause of prematurity, baby rocking recliner and baby walker usage were negative factors, while play in a sitting position, opportunity to move on a traditional mat and sleep mattress were positive factors contributing to the segmental control of the trunk. (4) Conclusions: the experience of sitting on different surfaces and an opportunity to sit without support during the child rearing period from age of 4 months corrected could be positive factors associating with the segmental trunk control in moderate-to-late premature infants.

## 1. Introduction

Independent sitting capability is a motor landmark, having a downstream effect on an infant’s motor development [1,2]. The age of sitter infants varies, ranging from 3.8 to 9 months in infants [3]. The essential achievement of independent sitting requires an infant’s ability to anticipate and grade muscle responses to the trunk to counteract gravitational torque. The neuromuscular coordination of the trunk occurs due to continuous and progressive organisation of the head, thoracic, lumbar and sacral segments. At this age, range postural control is developed in parallel. Saavedra and colleagues (2012) investigated postural control across multiple trunk segments in infants aged from 3 to 9 months via a prospective longitudinal study. They found that the progression of trunk control in sitting was in a cephalocaudal direction in segmental parts. Therefore, postural control during sitting should be evaluated in segments rather than in a single unit [4].

The ability of preterm infants to sit independently tends to arrive slower than in full-term infants [5,6,7,8]. Previous studies have investigated the developmental changes of segmental trunk control in a sitting posture in preterm infants [8,9]. They found a significantly high correlation between the lower thoracic level of trunk control and sitting ability at 7 months in moderate-to-late preterm infants [9]. While Pin and colleagues in 2020 found significant fair correlations between gross motor movement, such as prone, sitting and standing at 8 and 12 months of age and the SATCo, there was non-significant correlation at 4 months of age in preterm infants born at less than 32 weeks of gestation with biological risk [10]. The different levels of trunk control in segments result in different ages of sitting onset. Moderate preterm infants at 3 months displayed 12% abnormal motor development [7]. Besides, preterm infants at 6 months old demonstrated a lower sitting ability measured by the Alberta Infant Motor Scale (AIMS) than those with a full-term [6]. Low-risk preterm infants aged from 4 to 11 months corrected age showed a struggle to hold their trunk up against gravity for exploration and to achieve daily activities during sitting in the home environment [5]. Furthermore, the extremely preterm infants in a previous study could sit independently at the mean age of 8 months corrected, which indicated sufficient control of the trunk segments ranging from upper thoracic to lower lumbar levels [10].

Apart from biological factors, the onset age, rate and motor development sequence could be influenced by cultural contexts, such as child rearing and infant experience or interaction with mothers. Early motor skill acquisition was significantly advanced if infants received direct support with augmented practice enrichment [11]. An infant’s experience and environment at various times would lead to different onset of movement and posture control [12]. Karasik and colleagues’ cross-cultural study from six countries worldwide indicated that age onset of independent sitting at 5 months in typically developing infants has remarkable variation. They recommended that sitting at an earlier age and stability of sitting is attributable to the culture of child rearing, sitting experience and sitting environment [13]. Moreover, maternal education [14], performing reach for objects [15], playing in a sitting posture [16] or in the prone posture while awake [15], and equipment use [17] were significant factors affecting gross motor development. To comprehend an infant’s motor development with the non-conspicuous sign of neurological dysfunction like moderate-to-late preterm infants, series assessment is suggested. Darrah and colleagues recommended that longitudinal assessments are appropriate to reflect actual motor abilities [18]. Pin and colleagues’ longitudinal study examined the development of segmental trunk control between 31 extremely preterm infants and 30 full-term infants aged from 4 to 12 months. The preterm infants generally had a delay in developing the same level of segmental trunk control in sitting compared with their full-term peers. This previous study confirms that the development of segmental trunk control of extremely preterm infants was significantly delayed compared with that of full-term infants [8]. Identification of the contributions of the factors and segmental trunk control during sitting is rare. Only a previous cross-sectional study of the contribution of segmental trunk support to motor skill acquisition by Duncan et al. (2018) found that the varied levels of parent-holding of the infant’s segmental trunk in sitting and standing influenced the proficiency of gross motor skills in full-term infants aged 1 to 8 months [19].

Research on longitudinal assessment of the contribution of biological and child rearing factors to segmental postural control during sitting development in moderate-to-late preterm home-raised infants is still limited. Investigation of segmental trunk control factors during sitting development via a longitudinal design is therefore needed [13]. The objective of this study was to investigate the factors affecting segmental postural control in sitting from the age of 4 months to the age of independent sitting attainment in moderate-to-late preterm infants.

## 2. Materials and Methods

### 2.1. Participants

The parents or guardians-infant dyads were simply selected in every second order from the name list that had been provided by the 26-district health promoting hospitals within a range of 50 km from Khon Kaen University. Participants and their families were recruited if infants were born at 32–36 weeks of gestation and able to join the study when aged 4 months corrected age for the first data collection if the infants had stable health condition. Parents or guardians-infant dyads were excluded if their infant had seizure or visual or hearing impairment, congenital abnormality, major brain damage such as cerebral palsy, periventricular leukomalacia more than Grade I [20] and intraventricular hemorrhage more than Grade II [21] or had a neonatal intensive care unit stay of more than 17 days. Parents or guardians who gave interviews were recruited if they took care of their child for at least eight hours per day for at least 3 days per week, were able to communicate in and understood Thai, were willing to participate and stay with their child and were able to provide child rearing during the period of data collection.

The sample size of the current study was calculated from data of 33 preterm infants (no published pilot data) using the mean values of segmental trunk control at 4 months (μ_2_) until the age of independent sitting (μ_1_). The sample size of this study was then calculated using the following formulas. The number of infants with prematurity = $\left[2{\left(Z_{\alpha }+Z_{\beta }\right)}^{2}\left(1+\left(T_{n}-1\right)\rho \right)\right]$/$[T_{n}[\left({µ}_{1}-{µ}_{2}\right)/\sigma {]}^{2}]$ [22]. The Zα2 was set at 1.96. Effect size (μ_1_ − μ_2_)/σ was 0.77. $T_{n}$ was the maximal timepoint of the study. The significant value of 95% CI and 80% power was calculated to determine the number of participants. ρ is the assumed correlation (0.3) of the repeated measures. Therefore, the study needed to recruit at least 41 participants for statistical analyses in this prospective analytical study [23]. Thirty-three participants from the pilot study were included in the current study.

### 2.2. Measurement Instruments

#### 2.2.1. Segmental Assessment of Trunk Control (SATCo)

The SATCo was developed by Butler and colleagues in 2010 [24]. The test shows a good inter-rater reliability (ICC = 0.84) and excellent intra-rater reliability (ICC = 0.98) [24] in typically developing children and children with neurological conditions.

The SATCo assesses 7 segments of trunk control in the cephalocaudal direction while the assessor manually supports the trunk from the shoulder girdle to the pelvic segment. The 7 levels of trunk control include head, upper thoracic, middle thoracic, lower thoracic, upper lumbar, lower lumbar, and full trunk controls. The infant is in a seated position on a customised bench with a secure strap around the pelvis. The assessor’s hands are placed horizontally around the infant’s trunk. The test is started in a top-down sequence, supporting from shoulder to pelvic regions and finally with no support. The test continues with lowering of support until the child clearly cannot maintain posture. Each segment is assessed with the three conditions of (a) static, (b) active and (c) reactive control. The static postural control is credited when the infant can maintain a neutral posture at different levels of manual support. The active control is the ability to maintain the body in a neutral posture while the infant is stimulated to turn the head to either side and return to the midline. The reactive control is finally credited if the infant is able to maintain a neutral posture during an external perturbation.

The result is recorded as either an absence or presence. The “NT” is given when infants are not ready for the test. Numerical values were allocated for each segmental trunk control in the case that infants were able to show their segmental trunk control in each test condition. For the static and active condition, 1 is for head control, 2 for upper thoracic control, 3 for mid thoracic control, 4 lower thoracic control, 5 for upper lumbar control, 6 for lower lumbar control and 7 for full trunk control, while the reactive condition starts from 2 to 7 as described above. The reactive condition is not tested at the head segment [24,25].

#### 2.2.2. The Structured Questionnaire

Parents or guardians were face-to-face interviewed using the structured questionnaire. There are two parts to the questionnaire. Data on part one were collected at the first time of data collection including the age of parents (years), pregnancy complications, history of abortion, marital status, family size and level of parental education. Part two was collected regarding the socioeconomic and environmental diversity at the corrected age from 4 months to the age of attaining independent sitting. This part consisted of parental occupation, main caregiver, parental income, and breastfeeding, equipment used when child rearing in various positions (i.e., lying position such as sleep mattress, traditional mat, adult bed, crib; semi-reclined position such as baby hammock, baby rocking recliner, car seat; sitting with support and without support such as baby walker with wheels, high chair, baby seat, belt carried in arm or lab, exercaucer and playpens), the favourite play position (i.e., supine, side, manual carrying, sit, prone, creeping), and frequency of exposure to various environment resources (i.e., never, occasionally, often, always).

#### 2.2.3. Ethical Clearance

The ethical approval to perform this study was obtained by the Khon Kaen University Ethics Committee for Human Research on the basis of the Declaration of Helsinki and the ICH Good Clinical Practice Guideline (Institutional Review Board number: IRB00008614, protocol ID no.: HE622153, 10 July 2019). The research protocol of this study was renewed from the Khon Kaen University Ethics Committee for Human Research on the basis of the Declaration of Helsinki and the ICH Good Clinical Practice Guideline (Institutional Review Board number: IRB00001189, 30 June 2020). Data collection was performed from July 2019 to August 2020 at each infant’s home. The researcher made appointments to collect the data after the parents had given their informed consent to participate voluntarily, and allowed their infants to be assessed for trunk control while sitting. The data were anonymised in order to not reveal patients’ identities, and analysis was conducted in a way in which the final results could not be linked to individual patients.

### 2.3. Procedure

Demographic and vaccination data of preterm infants were recorded from the personal health booklet. Parents were face-to-face interviewed using the structured questionnaires about their parental characteristics and the environmental diversity. Thereafter, an appointment was made for the assessment of segmental trunk control during sitting.

On the appointment date, the physical growth consisting of body weight and body height were measured. The SATCos were performed while the infants were alert and their segmental trunk control was assessed on a monthly basis from the corrected age of 4 months until infants had attained independent sitting. Independent sitting in this study is defined as the ability of infants to sit up straight with the head erect without hands to balance or support the position momentarily for at least 10 s [24]. We asked parents or guardians to note the date of independent sitting attainment in the logbook recording (parents/guardians note) and call to inform the researcher. In order to verify the ability of independent sitting according to the operational definition as mentioned, the independent sitting of infant has been tested by the researcher within 5 days after the parents or guardians noted and reported. Subsequent assessments occurred on the same date (plus or minus 5 days) of every month. Distinctive sounds and a brightly coloured toy were used in order to induce the movement of the infant. An experienced paediatric physical therapist with 3 years of clinical experience learned how to perform of the SATCo measurement via the online method for 1 month [26]. Then she was trained how to use the SATCo by an expert with more than 5 years clinical experience in paediatrics and familiar with the SATCo and practiced performing the test for 6 months before data collection to ensure sufficient skills in administration of the tool. Intra-rater reliability of the SATCo were performed via the randomised video recordings on two separate days with a one-month interval in 25 preterm infants aged four, six and nine months who did not participate in the main study.

Parents were also interviewed monthly about environmental factors and a logbook was given to each family to record (1) the favourite play position, (2) equipment used at various positions and (3) frequency of exposure to various environments.

### 2.4. Data Analysis

Descriptive statistics were used to analyse demographic data of preterm infants and parents. The outcome of the study was the trunk segment development; the ordinal score of SATCo defined as the level of trunk segment at each condition: static, active, and reactive control were repeated measure monthly. The development of static, active and reactive condition of SATCo between times in each condition was observed. In this study, related factors were measured as a discrete variable that was independent and included multiple repeats during overall time and in each month (at least 3 months up to 6 months). The generalised estimating equation (GEE) model was used to determine the factors contributing the trunk segment development with a linear distribution. The association between all conditions of SATCo and related factors was estimated in two steps. We conducted univariate GEE analyses to determine crude associations between all conditions of SATCo scores and related biological and child rearing factors using the Wald Chi-Squared. Forty-two infants were recruited and the number of data collection times ranged from a minimum of 3 to a maximum of 6 times. Therefore, in the first step of data analysis, 176 data sets were included in the univariate analyses for variable selection and set the *p*-value at < 0.25. Significant variables from the first step had been included in the second step of the GEE model to consider significant related factors used as the independent variables. The significance level was set at *p* < 0.05 with a 95% confidence interval for statistical tests. To allow for time varying covariates, we put all significant factors correlated to the SATCo scores with robust standard errors. The unit of analysis was infant-months. The Statistical Package for Social Sciences (SPSS) version 17 (SPSS Inc., Chicago, IL, USA) was used to carry out all statistical analysis.

## 3. Results

The intra-rater reliability (ICC (3,1)) of the SATCo was reported to be 0.936 (95% CI 0.860–0.971). Name lists of 58 moderate-to-late preterm infants were obtained from 26-district health promoting hospitals. Sixteen of 58 infants were not recruited due to 1 infant declining to participate and 15 families moving house. Data of the remaining 42 infants were then collected and analysed. The mean age of first assessment was 4 months and 5 days corrected age. The mean age of attaining the independent sitting of infants in this study was 7 months and 6 days (SD, 1 month 10 days) corrected age.

Table 1 shows mean (SD), and range of neonatal characteristics of preterm infants at inclusion. There were 28 boys (66.7%) and 14 girls (33.3%). Average gestational age of preterm infants was 34.5 (1.6) weeks. Distribution of infants from different gestational ages were 11 infants at 32 weeks, 8 infants at 34 weeks, 8 infants at 35 weeks and 15 infants at 36 weeks. Nine infants were born preterm with unknown reasons. Thirty-three infants had cause of prematurity including antepartum hemorrhage, premature rupture of membrane, intrauterine growth restriction and eclampsia. Thirty-two (76.2%) infants had a low birth weight. According to the Standard Intrauterine Growth Curve of Thai Neonates, 19 (45.2%) infants were small for gestational age, 19 (45.2%) were appropriate for gestational age and 4 (9.6%) were large for gestational age. Neonatal complications were mild bronchopulmonary dysplasia (*n* = 13, 31%), neonatal jaundice (*n* = 9, 21.4%) and G6PD deficiency (*n* = 2, 4.8%). Furthermore, most infants (36 of 42) dwelled with their parents and 6 infants with single mothers.

Table 2 presents characteristics of parents. The mean maternal age was 27.5 (7.1) (range = 15–42) years. Nine mothers were adolescents. The mean age of fathers was 28.1 (7.4) (range 16–44) years. Twenty-five mothers (59.5%) had pregnancy complications including intrauterine growth restriction, antepartum hemorrhage, premature rupture of membrane, preeclampsia and hypertension. Fourteen mothers had a history of abortion.

Table 3 shows the related factors at 4 months to the age of independent sitting attainment consisted of play in sitting position, equipment used while awake including play in sitting position, traditional mat, sleep mattress, belt for carrying in arm/lab, playpens, baby rocker recliner and baby walker. The different numbers of infants at 7, 8, and 9 months were due to some infants achieving independent sitting at 7 months, so only the remaining infants still participated in the data collection.

Table 4 shows the median (range) of the SATCo scores in all three conditions at age 4 months to the age of attaining independent sitting. The median SATCo score of each condition increased slightly in accordance with age. At each age, the median SATCo score of the reactive condition was the lowest compared with those in the static and active conditions.

Table 5 shows univariate GEE analyses of related factors on the SATCo in premature infants identified the following significant factors for static SATCo score: cause of prematurity (χ^2^ = 11.39, *p* < 0.001); small for gestational age (χ^2^ = 0.29, *p* = 0.589); play in sitting position (χ^2^ = 144.52, *p* < 0.001); traditional mat (χ^2^ = 7.81, *p* = 0.005); sleep mattress (χ^2^ = 25.30, *p* = <0.001); belt for carrying in arm/ lab (χ^2^ = 2.47, *p* = 0.116); playpen (χ^2^ = 2.75, *p* = 0.097); baby rocking recliner (χ^2^ = 6.43, *p* = 0.011) and baby walker (χ^2^ = 47.61, *p* < 0.001) (Table 5).

Factors analysed by univariate GEE were introduced into the GEE model with adjustment for all the analysed parameters. Statistically significant related factors for the active SATCo included the following: cause of prematurity (χ^2^ = 1.18, *p* < 0.278); small for gestational age (χ^2^ = 0.19, *p* = 0.665); play in sitting position (χ^2^ = 119.27, *p* < 0.001); traditional mat (χ^2^ = 12.29, *p* = < 0.001); sleep mattress (χ^2^ = 35.54, *p* = < 0.001); belt for carrying in arm/lab (χ^2^ = 1.92, *p* = 0.116); playpen (χ^2^ = 2.49, *p* = 0.114); baby rocking recliner (χ^2^ = 6.14, *p* = 0.013) and baby walker (χ^2^ = 43.68, *p* < 0.001) (Table 5).

The univariate GEE was used to verify the association between the reactive SATCo and related factors. The variables that were considered to be included in the GEE model comprised of: cause of prematurity (χ^2^ = 4.14, *p* < 0.042); small for gestational age (χ^2^ = 0.75, *p* = 0.385); play in sitting position (χ^2^ = 122.93, *p* < 0.001); traditional mat (χ^2^ = 9.13, *p* = < 0.001); sleep mattress (χ^2^ = 40.27, *p* = < 0.001); belt for carrying in arm/lab (χ^2^ = 2.84, *p* = 0.092); playpen (χ^2^ = 2.16, *p* = 0.142); baby rocking recliner (χ^2^ = 4.38, *p* = 0.013) and baby walker (χ^2^ = 57.62, *p* < 0.001) (Table 5).

Table 6 reports the factors associated with the segmental postural control among three conditions from 4 months to the age of independent sitting which were analysed by GEE. The model showed that the positive associated factors of segmental postural control were play in sitting position and sleep mattress for all conditions and traditional mat for static and active conditions (*p* < 0.05). Whereas the negative associated factors were cause of prematurity at static condition, baby rocking recliner and baby walker for all conditions (*p* < 0.05).

## 4. Discussion

The purpose of this prospective study was to investigate the contribution of related factors on the segmental trunk control in sitting from the age of 4 months to the age of independent sitting attainment in moderate-to-late preterm infants. The levels of trunk control segment were estimated by the SATCo scores. The findings support that the environment during child rearing practice is a factor associated with segmental postural control. This study demonstrates the cause of prematurity and child rearing factors at 4 months associated with the segmental postural control in moderate-to-late preterm infants. Results found that the usage of a baby rocking recliner and baby walker were negative factors, while an experience of floor sitting on a traditional mat and sleep mattress were positive factors contributing to SATCo scores during the development of the sitting milestone. However moderate-to-late premature infants in this study did not show delayed sitting onset. The explanation could be due to the design and objective of this longitudinal study, therefore we cannot determine the effect of positive and negative factors on SATCo scores.

Previous studies have provided evidence that late preterm infants displayed onset of sitting ability and achieved the same level of segmental postural control later than full-term peers [8,9,27]. Sato and Tudella (2018) reported that 6 to 8 months of 20 late preterm infants were delayed in controlling the trunk compared with 36 full-term assessed by the SATCo. Mainly in independent sitting, 9.09% of all full-term displayed total trunk control at 7 months, and 71.42% of full-term showed total trunk control at 8 months [27]. Achieving trunk control at the mid thoracic level of full-term has been linked to independent sitting at 6 months, whereas this association appears in preterm infants at 7 months [9]. Pin and colleagues found in 2020 that the extremely preterm infants started to sit without hand support at 8 months, with their segmental trunk control ranging from the upper lumbar to the lower lumbar levels [10]. The abilities of segmental trunk control in these extremely preterm infants were probably due to the extensor and flexor muscle patterns’ asymmetry.

The present study did not examine the segmental trunk control of full-term infants, which is a limitation of our interpretation of comparison of control the segmental trunk between the moderate-to-late preterm and full-term peers. Our investigations found that the development pattern of segmental trunk control that emerges during sitting is consistent with previous evidence [8,9,27]. Approximately 55% of 42 preterm infants could sit independently at a mean age of 7 months. Although this study shows the gradual acquisition of the segments of the trunk, they could not accomplish full trunk control at the age of defined independent sitting. This could be due to the onset of independent sitting in this study being defined as an ability to sit without support momentarily for at least 10 s. Future study could assess the segmental trunk control of preterm infants longitudinally, until they fully obtain independent sitting skills compared with typical full-term infants.

Results of segmental postural control in infants could be attributed to the interaction of many factors. The affecting factors to the SATCo scores found during the perinatal period were the causes of prematurity, such as intrauterine growth restriction (IUGR). A previous population-based cohort study in France found that perinatal complications, such as mothers with IUGR and gestational hypertension, were likely to have infants with preterm births. The preterm infants also frequently display later motor development compared to those who were born full term [28]. The low gross motor development was reported to be related to low gestational age at birth of the infant. Moreover, preterm infants in Brazil who experienced IUGR had significantly lower averages of gross motor development from 13 to 30 months [29]. Another factor affecting motor development of premature birth from the literature [30] was infants who were born small for their gestational age. Hediger reported that moderate-to-late preterm infants with 33 to 36 weeks of gestation aged 2 to 47 months born small is primarily attributable to the delay in gross motor-social developments. Moreover, preterm infants born small had explicit delay gross motor and motor-social problems in their developments more than full-term infants born small [30]. Almost half (*n* = 19, 45.2%) of participants in our study were small for gestation, however the univariate analysis found no statistical significance of this factor.

Child rearing practices and contextual factors critically impact the proficiency of sitting during childhood [11,13]. Karasik and colleagues’ study [13] reported the sitting behavior of full-term infants at 5 months, and found that 11 (92%) Cameroonian infants whose mothers placed them in “tripod” ground sitting for an average of 9 min and sitting on high adult furniture for an average of 13 min could sit independently, while the ability to sit independently occurred in only three (25%) Argentinian infants due to sitting practices for less than 1 min. Italian infants had daily routines spending time in mother’s arms, which led to an inability to sit independently. Researchers described that a few minutes of unsupported sitting and moving the Italian infant’s body per day distributed over a week is insufficient to practice postural control across various places [13]. On the contrary, African and Caribbean infants whose mothers hung them close to their body during daytime working could stimulate the infants upright postural control [11].

Experience of movement and exploration on the floor with variation in surface provides valuable augmented practice to encourage the development of segmental trunk control in preterm infants. The present study found that spending time on a traditional mat while awake was significantly associated with segmental control in static and active conditions (Table 4). Moreover, using the sleep mattress was associated with infants’ segmental trunk control in 3 conditions (Table 4). In the context of the current study, the term “traditional mat” refers to a reed mat (craft) with slimness and various sizes covering the floor. The sleep mattress’ thickness and softness would create an unstable wobbling situation, while infants need to maintain their posture in upright sitting. Infants who experience sitting from firm to wobbly surfaces on the floor can experience a challenging movement. This result may support previous studies indicating increased maturation, learning and motor skill acquisition from an environment with altering sitting experiences in infancy [31,32]. Several studies have also highlighted that sitting proficiency could be linked to parental encouragement [13,17,33]. The previous study shows that Kenyan infants with experience of sitting on different floor surfaces and high adult furniture could sit stably at an earlier age than American infants living in cultures using trunk support to the child using equipment [13].

The infants who received long duration of trunk support from equipment such as a baby walker are negatively related to motor ability [34,35]. Bezgin and Colleagues in 2020 found that using a baby walker affected a delay in trunk control of 29 typical developing infants compared with 19 age-matched peers who did not use a baby walker with the average age of 10 months corrected age. They also reported that the duration of use of baby walkers, approximately 2 months, were associated with lower AIMS percentage scores (*p* < 0.05) and SATCo scores in the reactive and total subscales (*p* < 0.05) than those of the infants not using the baby walker. The previous study explained that the restrictions on free movement due to restriction from the rigid structure of the baby walker give less opportunity to transfer weight to their trunk, hip and lower extremities, resulting in a lack of improving the balance in space for weight shifting and controlling their antigravity posture in sitting positions [35].

The previous study suggested that infants should have an opportunity or experience to move on their belly [11] or frequently sit without external support [13] in safe circumstances during their awake time. We found that using a baby rocking recliner is also negatively associated with the segmental control in all conditions of SATCo. The result was in line with the systematic review that delay of the head control, rolling and bimanual use was related to parents continuing to put their infants in supine equipment [36]. Duncan and colleagues in 2018 also found that infants who were held at a higher level on their trunk than the infant’s SATCo level were more likely to have developmental delays on the AIMS. [19].

Although we found significant results, the study contained some limitations. We did not examine the segmental trunk control in full-term peers longitudinally, and there were no cut-off scores, so this study could not determine whether the infants in our study showed delays in their longitudinal SATCo scores. Furthermore, we did not receive data of the duration and frequency of activity in all movement experiences. A future study should conduct randomised controlled studies investigating the effect of sitting surface, frequencies and durations of movement or baby equipment usage on the SATCo score during their awake time, and this could be reported by caregivers or parents using a standardised tool such as the Daily Activities of Infant Scale [5].

## 5. Conclusions

The present study investigated the factors affecting segmental postural control in sitting from the age of 4 months to the early onset of independent sitting attainment via a longitudinal study in moderate-to-late preterm infants. Biological and child rearing circumstances of moderate-to-late preterm infants were found to impact with a series of significant associations to their segmental postural control. The experience of or opportunity to perform sitting without support is an essential basis for development of trunk segments in premature infants.

## Figures and Tables

**Table 1:** Characteristic of all infants (*n* = 42).

Demographics Data	Mean (SD)	Range
Birth weight (g.)	2206.7 (4439.4)	1330–3092
Birth length (cm.)	47.6 (1.9)	44–51
Birth head circumference (cm.)	30.6 (1.5)	29–34
The Apgar Score at 5 min	9.3 (0.5)	8–10
Gestational age	34.5 (1.6)	32–36

**Table 2:** Parental characteristics.

Demographic Data	*n* (%)
**Pregnancy complications**	
Anemia	4 (16.0)
Gestational diabetes mellitus	6 (24.0)
Hypertension	7 (28.0)
Intrauterine growth restriction	10 (40.0)
Preeclampsia	9 (36.0)
Thalassemia	1 (4.0)
Antepartum hemorrhage	10 (40.0)
Premature rupture of membrane	10 (40.0)
**Marital status**	
Married	36 (85.7)
Divorced	6 (14.3)
**Family size**	
A single family	9 (21.4)
An extended family	33 (78.6)
**The education of mother**	
Primary school	1 (2.4)
Secondary school	16 (38.1)
High school	12 (28.6)
Vocational college	2 (4.8)
Bachelor’s degree	10 (23.8)
Master’s degree	1 (2.4)
**The education of father**	
Primary school	0 (0)
Secondary school	16 (38.1)
High school	14 (33.3)
Vocational college	3 (7.1)
Bachelor’s degree	8 (19.0)
Master’s degree	1 (2.4)

Note: Data present counts (percentage in parenthesis).

**Table 3:** Numbers (%) of infants using different equipment from 4 months to the age of independent sitting.

Factors	4 Months	5 Months	6 Months	7 Months	8 Months	9 Months	Independent Sitting
	(*n* = 42)	(*n* = 42)	(*n* = 42)	(*n* = 35)	(*n* = 12)	(*n* = 3)	(*n* = 42)
Play in sitting position	2 (4.8)	5 (11.9)	11 (26.2)	27 (77.1)	9 (75.0)	3 (100.0)	38 (90.5)
Traditional mat	12 (33.3)	22 (52.4)	24 (57.1)	23 (65.7)	6 (50.0)	1 (33.3)	25 (59.5)
Sleep mattress	37 (88.1)	41 (97.6)	33 (78.6)	25 (71.4)	6 (50.0)	2 (66.7)	32 (76.2)
Belt for carrying in arm/lab	25 (59.5)	18 (42.9)	13 (31.0)	12 (34.3)	6 (50.0)	2 (66.7)	16 (38.1)
Playpens	2 (4.8)	4 (9.5)	5 (11.9)	6 (17.1)	5 (41.7)	3 (66.7)	8 (19.0)
Baby rocker recliner	17 (40.5)	17 (40.5)	23 (54.8)	6 (17.1)	1 (8.3)	0 (0.0)	7 (16.7)
Baby walker	12 (28.6)	20 (47.6)	29 (69.0)	29 (82.9)	11 (91.7)	3 (100.0)	37 (88.1)

Note: Data present counts (percentage in parenthesis).

**Table 4:** The SATCo scores from 4 months to the age of independent sitting.

Corrected Age of Prematurity	SATCo Conditions	Level of Trunk Segment	Median	Range
4 months	Static	Mid thoracic	3	2 to 5
	Active	Upper thoracic	2	1 to 4
	Reactive	Upper thoracic	2	1 to 4
5 months	Static	Lower thoracic	4	3 to 6
	Active	Mid thoracic	3	2 to 5
	Reactive	Mid thoracic	3	2 to 4
6 months	Static	Upper lumbar	5	4 to 7
	Active	Lower thoracic	4	3 to 6
	Reactive	Mid thoracic	3	3 to 6
7 months	Static	Full trunk	7	5 to 7
	Active	Upper lumbar	5	3 to 7
	Reactive	Lower thoracic	4	3 to 6
8 months	Static	Full trunk	7	5 to 7
	Active	Upper lumbar	5	4 to 6
	Reactive	Upper lumbar	5	3 to 6
9 months	Static	Full trunk	7	7 to 7
	Active	Lower lumbar	6	5 to 6
	Reactive	Upper lumbar	5	4 to 6
Age of independent sitting	Static	Full trunk	7	5 to 7
	Active	Upper lumbar	5	4 to 7
	Reactive	Upper lumbar	5	4 to 6

Numbers are median and range of SATCo (the Segmental Assessment of Trunk Control) scores, and also represent the level of trunk segment: 1 = head; 2 = upper thoracic; 3 = mid thoracic; 4 = lower thoracic; 5 = upper lumbar; 6 = lower lumbar; 7 = full trunk control.

**Table 5:** Univariate analysis, the Wald Chi-Squared (*p*-value) of factors associating with the segmental trunk control in 3 conditions of SATCo.

Factors	Conditions of SATCo					
	Static		Active		Reactive	
Cause of prematurity	11.39	(<0.001) *#	1.18	(0.278)	4.14	(0.042) *#
Small for gestational age	0.29	(0.589)	0.19	(0.665)	0.75	(0.385)
Play in sitting position	144.52	(<0.001) **#	119.27	(<0.001) **#	122.93	(<0.001) **#
Traditional mat	7.81	(0.005) *#	12.29	(<0.001) **#	9.13	(<0.003) *#
Sleep mattress	25.30	(<0.001) **#	35.54	(<0.001) **#	40.27	(<0.001) **#
Belt for carrying in arm/lab	2.47	(0.116) #	1.92	(0.166) #	2.84	(0.092) #
Playpen	2.75	(0.097) #	2.49	(0.114) #	2.16	(0.142) #
Baby rocking recliner	6.43	(0.011) *#	6.14	(0.013) *#	4.38	(0.013) *#
Baby walker	47.61	(<0.001) **#	43.68	(<0.001) **#	57.62	(<0.001) **#

* Significant correlation (*p* < 0.05); ** significant correlation (*p* < 0.01); # significant correlation (*p* < 0.25).

**Table 6:** Estimated associating factors with the segmental trunk control in 3 conditions.

Factors	Conditions of SATCo								
	Static			Active			Reactive		
	β	95%CI	*p*-Value	β	95%CI	*p*-Value	β	95%CI	*p*-Value
Cause of prematurity	−0.495	4.00 to 5.08	<0.001 *	−0.202	0.52 to 0.11	0.206	−0.227	−0.50 to 0.05	0.102
Play in sitting position	1.436	−0.81 to −0.18	0.002 *	1.409	1.04 to 1.78	<0.001 **	1.430	1.10 to 1.76	<0.001 **
Traditional mat	0.487	0.15 to 0.83	0.005 *	0.487	0.12 to 0.86	0.010 *	0.290	0.00 to 0.58	0.050
Sleep mattress	0.542	0.19 to 0.84	0.003 *	0.557	0.21 to 0.91	0.002 *	0.490	0.18 to 0.81	0.002 *
Belt for carrying in arm/lab	0.126	−0.23 to 0.48	0.487	0.124	−0.16 to 0.41	0.399	0.146	−0.11 to 0.41	0.268
Playpens	0.181	−0.10 to 0.47	0.210	0.309	−0.20 to 0.82	0.230	0.273	−0.18 to 1.40	0.237
Baby rocking recliner	−0.519	−0.77 to −0.27	<0.001 **	−0.271	−0.54 to −0.01	0.047 *	−0.264	−0.52 to −0.01	0.044 *
Baby walker	−0.476	−0.79 to −0.16	0.003 *	−0.439	−0.75 to −0.13	0.006 *	−0.449	−0.69 to −0.21	<0.001 **

Note: * Significant value (*p* < 0.05); ** Significant value (*p* < 0.001).
